# Divergence in Sex Steroid Hormone Signaling between Sympatric Species of Japanese Threespine Stickleback

**DOI:** 10.1371/journal.pone.0029253

**Published:** 2011-12-28

**Authors:** Jun Kitano, Yui Kawagishi, Seiichi Mori, Catherine L. Peichel, Takashi Makino, Masakado Kawata, Makoto Kusakabe

**Affiliations:** 1 Ecological Genetics Laboratory, National Institute of Genetics, Mishima, Shizuoka, Japan; 2 Department of Genetics, The Graduate University for Advanced Studies (Sokendai), Mishima, Shizuoka, Japan; 3 PRESTO, Japan Science and Technology Agency, Honcho Kawaguchi, Saitama, Japan; 4 Graduate School of Life Sciences, Tohoku University, Sendai, Miyagi, Japan; 5 Program in Developmental Biology, Baylor College of Medicine, Houston, Texas, United States of America; 6 Biological Laboratory, Gifu-keizai University, Ogaki, Gifu, Japan; 7 Division of Human Biology, Fred Hutchinson Cancer Research Center, Seattle, Washington, United States of America; 8 Atmosphere and Ocean Research Institute, The University of Tokyo, Kashiwanoha, Chiba, Japan; Ecole Normale Supérieure de Lyon, France

## Abstract

Sex steroids mediate the expression of sexually dimorphic or sex-specific traits that are important both for mate choice within species and for behavioral isolation between species. We investigated divergence in sex steroid signaling between two sympatric species of threespine stickleback (*Gasterosteus aculeatus*): the Japan Sea form and the Pacific Ocean form. These sympatric forms diverge in both male display traits and female mate choice behaviors, which together contribute to asymmetric behavioral isolation in sympatry. Here, we found that plasma levels of testosterone and 17β-estradiol differed between spawning females of the two sympatric forms. Transcript levels of *follicle-stimulating hormone-β* (*FSHβ*) gene were also higher in the pituitary gland of spawning Japan Sea females than in the pituitary gland of spawning Pacific Ocean females. By contrast, none of the sex steroids examined were significantly different between nesting males of the two forms. However, combining the plasma sex steroid data with testis transcriptome data suggested that the efficiency of the conversion of testosterone into 11-ketotestosterone has likely diverged between forms. Within forms, plasma testosterone levels in males were significantly correlated with male body size, a trait important for female mate choice in the two sympatric species. These results demonstrate that substantial divergence in sex steroid signaling can occur between incipient sympatric species. We suggest that investigation of the genetic and ecological mechanisms underlying divergence in hormonal signaling between incipient sympatric species will provide a better understanding of the mechanisms of speciation in animals.

## Introduction

Hormones mediate the regulation of diverse phenotypic traits [1], [2]. Therefore, differences in hormonal pathways between sympatric species can underlie divergence in traits important for adaptation [3] and reproductive isolation. Thus, hormonal studies are crucial for a better understanding of speciation mechanisms. For example, when sexual selection drives or promotes speciation [4], [5], it is particularly important to determine whether divergence in hormonal signaling regulates the expression of sexually dimorphic traits that contribute to reproductive isolation between species. Even when ecological adaptation plays a major role in speciation [6], [7], hormonal signaling could modulate the expression of adaptive traits that contribute to reproductive isolation. Although the ecological and genetic mechanisms of speciation have been extensively investigated during the last few decades [7], [8], [9], relatively little is known about the hormonal basis for speciation.

Sex steroids mediate the expression of sexually dimorphic or sex-specific traits important for behavioral isolation, such as courtship ornaments and female mate choice behaviors [10], [11], [12]. Because male ornaments and female mate choice behaviors are often sexually antagonistic, plasma levels of sex steroid hormones regulating these traits can also be sexually antagonistic [1], [13], [14]. For example, androgens are important mediators of the expression of male ornaments. Therefore, sexual selection should favor high androgen levels in males. By contrast, high androgen levels might be detrimental in females, because possession of exaggerated ornaments may be energetically costly or attract predators [4], [15], or because high androgen levels may suppress immune response [12], [16], [17], [18]. Therefore, the optimal values of sex steroid levels are likely to differ between the sexes. However, significant genetic correlations exist between males and females for sex steroid levels [13], [14], which can constrain the evolution of sexual dimorphism in sex steroid levels within species, as well as divergence in the magnitude of sexual dimorphism between closely related species. Understanding how the patterns of sex differences in steroid levels can diverge between closely related species will provide insight into the physiological mechanisms underlying speciation.

The threespine stickleback (*Gasterosteus aculeatus*) species complex is a great model for exploring the genetic and ecological mechanisms underlying phenotypic divergence and reproductive isolation between closely related species [19], [20], [21], [22]. Tremendous diversification of threespine stickleback has occurred during the last few million years and resulted in the evolution of multiple phenotypically and ecologically divergent forms, which are often reproductively isolated in sympatry [21], [23], [24]. Divergence in male display traits often contributes to reproductive isolation between sympatric forms [23], [25], [26], [27]. In sticklebacks, sex steroids not only regulate expression of sexually dimorphic traits [28], [29], [30], [31], [32], [33], [34], [35], but also suppress immune response [17]. These empirical data suggest that sex steroid levels may be sexually antagonistic in sticklebacks. Although sex differences in plasma levels of several sex steroids have been found in a European stickleback population [29], little is known about variation in sex steroid levels or in the magnitude of the sex differences between divergent stickleback forms.

In the present study, we investigated plasma sex steroid levels of nesting males and spawning females in a sympatric pair of Japanese threespine stickleback, comprising the Pacific Ocean and Japan Sea forms [26], [36]. These two forms diverged 1.5–2 million years ago when the Sea of Japan was geographically isolated from the Pacific Ocean. After the glacial recession, these two forms were brought into secondary contact. Although the two forms are currently sympatric in coastal regions of eastern Hokkaido, Japan, they are reproductively isolated, in part due to asymmetric behavioral isolation [26], [36], [37]. Asymmetric behavioral isolation is one of the isolating barriers between them. Pacific Ocean females virtually always mate with Pacific Ocean males, while Japan Sea females mate with males of both forms at similar frequencies [26], [36], [38]. Pacific Ocean female mate choice is based on divergence in male body size and male dorsal pricking behavior [36]. Pacific Ocean males and females are larger than Japan Sea males and females, and Pacific Ocean females prefer to mate with larger males. In addition, Pacific Ocean females also do not like the aggressive dorsal pricking of Japan Sea males.

**Figure 1:**
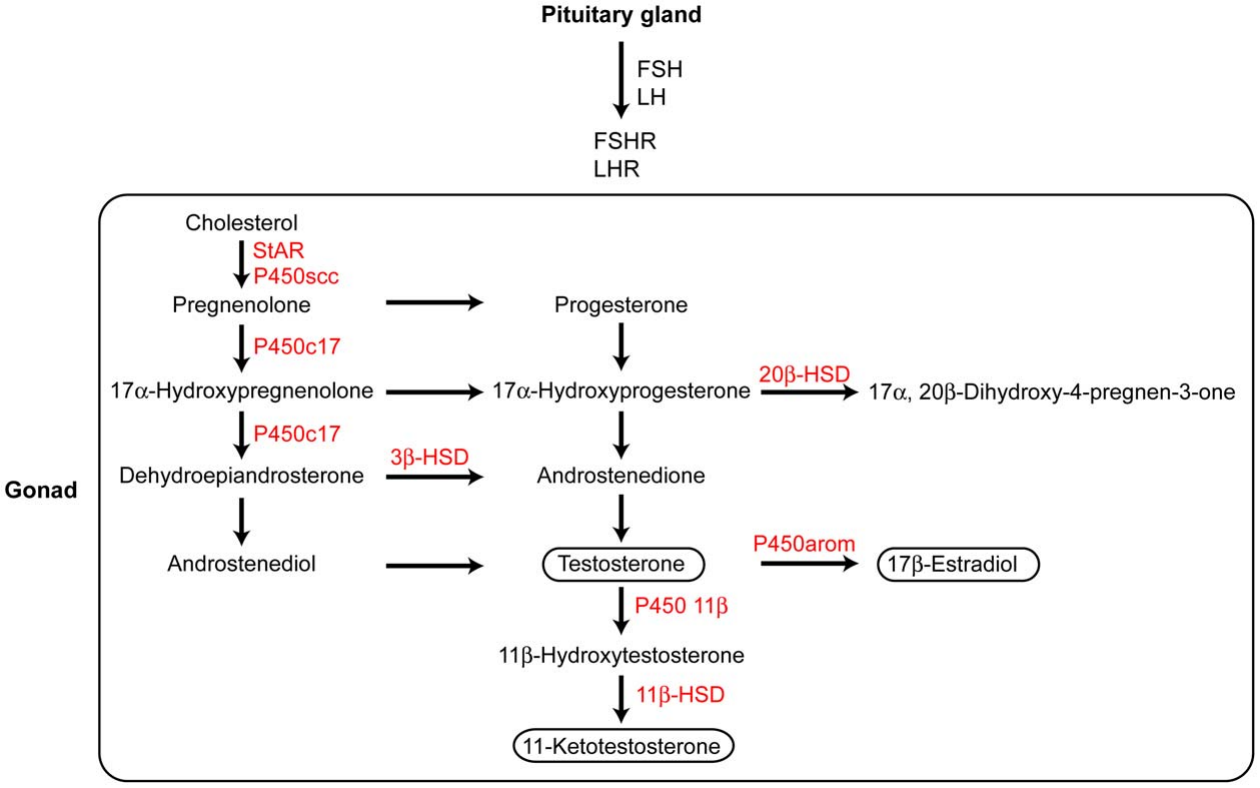
Proposed steroidogenic pathways. Because steroidogenic pathways in sticklebacks have not been elucidated, steroidogenic pathways in rainbow trout are shown here [46], [49], [50], [81]. Steroidogenic enzymes are shown in red. HSD, hydroxysteroid dehydrogenase; P450, cytochrome P450.

Previous genetic mapping in the Japanese sympatric pair revealed that divergence in sexually dimorphic traits mapped to the sex chromosomes [36]. This result is consistent with the theoretical prediction that sexual antagonism can be resolved by sex-linkage [39], [40], [41]. However, sex-linkage is not the only mechanism by which sexual antagonism can be resolved, but sex-specific transcriptional regulation is another important mechanism [42]. Sex steroid hormones are important mediators of sex-specific transcriptional regulation in vertebrates [13]. Sex steroid hormones are mainly secreted from the gonads (Fig. 1) and are regulated by pituitary hormones, such as luteinizing hormone (LH) and follicle-stimulating hormone (FSH) [31]. Thus, to gain further insight into the mechanisms underlying divergence in reproductive traits that are important for behavioral isolation between the Japanese sympatric sticklebacks, we compared sex steroid signaling among nesting males and spawning females of the Japanese sympatric pair.

## Results

### Patterns of sex steroid variation in the Japanese sympatric species pair

**Figure 2:**
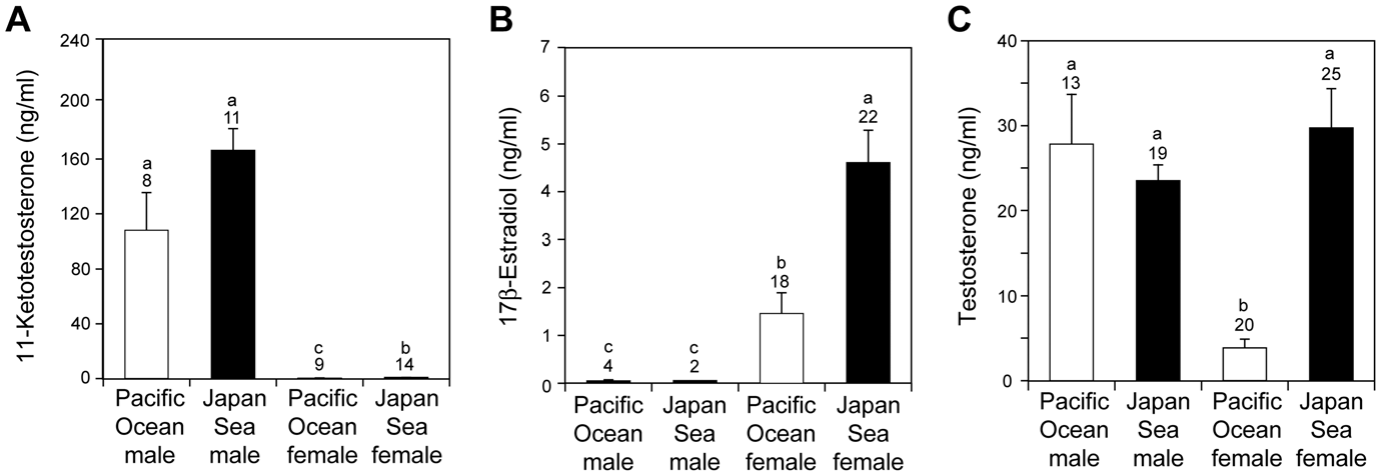
Divergence in sex steroid levels. **Comparison of plasma 11-ketotestosterone (A), 17β estradiol (B), and testosterone (C) between Pacific Ocean and Japan Sea forms of threespine stickleback. The sample size is shown above each column.** Lower case letters above the bars indicate samples that are significantly different from each other (Tukey HSD test after ANOVA).

Plasma 11-ketotestosterone levels were significantly higher in males than in females (Fig. 2A; *F*_1,38_ = 741.6, *P*<0.001) and were significantly higher in the Japan Sea form than in the Pacific Ocean form (*F*_1,38_ = 10.8, *P* = 0.002). No significant interaction between sex and form was found for plasma 11-ketotestosterone levels (*F*_1,38_ = 10.8, *P* = 0.660). 17β-Estradiol levels were significantly higher in females than in males (Fig. 2B; *F*_1,41_ = 80.3, *P*<0.001) and were significantly higher in the Japan Sea form than in the Pacific Ocean form (*F*_1,41_ = 28.0, *P*<0.001). No significant interaction between sex and form was found for plasma 17β-estradiol levels (*F*_1,41_ = 1.9, *P* = 0.174), although the small sample size available for males may have reduced the statistical power of this analysis (see the Materials and Methods for a discussion of the small sample size). Plasma concentrations of testosterone exhibited different patterns of sexual dimorphism between forms (Fig. 2C): significant interaction between sex and form was found (sex-by-form interaction, *F*_1,71_ = 18.8, *P*<0.001; the effect of sex, *F*_1,71_ = 14.6, *P* = 0.002; the effect of form, *F*_1,71_ = 33.1, *P*<0.001).

### Divergence in female sex steroids

**Figure 3:**
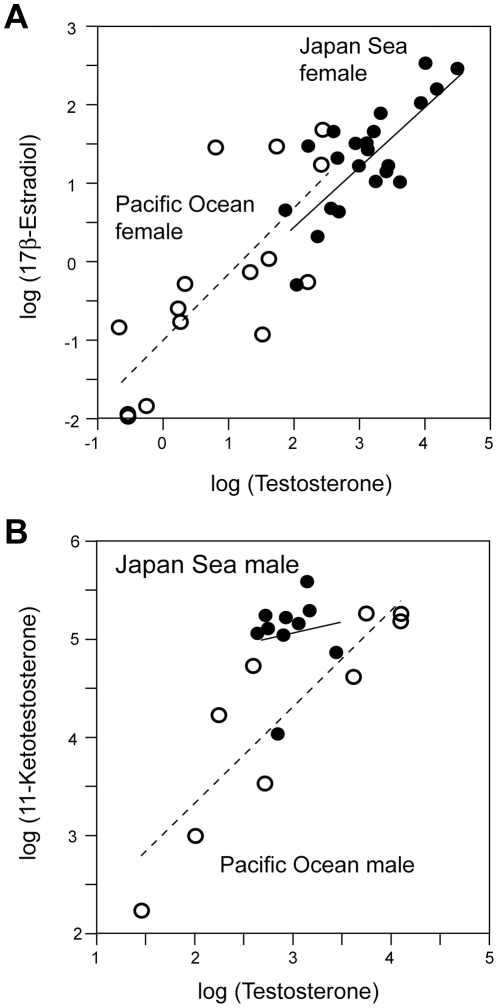
Correlations between plasma testosterone and other sex steroid hormone levels. Correlations between log-transformed plasma testosterone levels and log-transformed plasma 17β estradiol levels in females (A) and correlations between log-transformed plasma testosterone levels and log-transformed plasma 11-ketotestosterone levels in males (B). Solid lines and dotted lines indicate regression lines for the Japan Sea form and the Pacific Ocean form, respectively. Closed circles, Japan Sea form; open circles, Pacific Ocean form.

Females differed in the levels of all steroids examined, with Japan Sea females having significantly higher plasma concentrations than Pacific Ocean females (Fig. 2; 11-ketotestosterone, *F*_1,21_ = 7.39, *P* = 0.013; testosterone, *F*_1,41_ = 39.10, *P*<10^−6^; 17β-estradiol *F*_1,38_ = 27.05, *P*<10^−5^). Because 17β-estradiol is a product of testosterone [43] (Fig. 1), we investigated the relationship between plasma testosterone and 17β-estradiol levels (Fig. 3A). A significant relationship was found between testosterone and 17β-estradiol levels (ANCOVA, *F*_1,35_ = 95.06, *P*<10^−10^), but there was no significant difference between forms (ANCOVA, *F*_1,35_ = 0.01, *P* = 0.857) or interaction between form and testosterone (ANCOVA, *F*_1,35_ = 0.009, *P* = 0.923). These results suggest that the conversion of testosterone into 17β-estradiol in females does not differ between forms.

**Figure 4:**
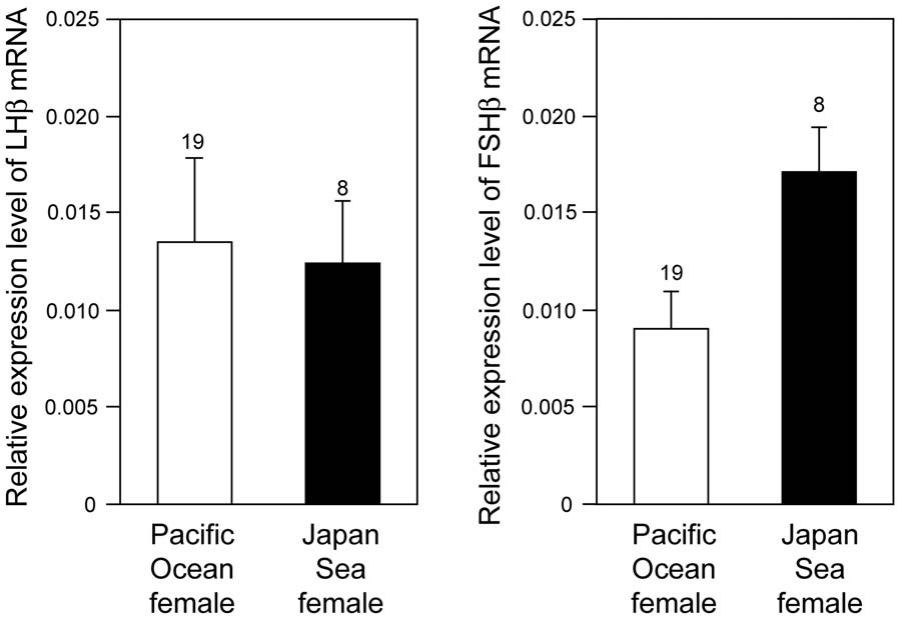
Relative expression levels of mRNA in the pituitary glands for *LHβ* and *FSHβ*, determined by qPCR. The sample size is shown above each column.

Because female sex steroid levels have diverged between the Japanese stickleback species, we also investigated whether there is divergence in the upstream signaling pathways for sex steroid production. The synthesis and secretion of sex steroid hormones from gonads is stimulated by pituitary glycoprotein hormones, follicle-stimulating hormone (FSH) and luteinizing hormone (LH) [44]. The functional FSH and LH hormones are dimers of a hormone-specific β-subunit (FSHββ) and an α-subunit that is shared with other pituitary glycoprotein hormones. Therefore, pituitary transcript levels of *LHβ* and *FSHβ* were compared. Transcript levels of *FSHβ* were significantly higher in Japan Sea females than in Pacific Ocean females (Fig. 4; ANOVA, *F*_1,25_ = 4.86, *P* = 0.037), but transcript levels of *LHβ* were not significantly different between forms (ANOVA, *F*_1,25_ = 0.04, *P* = 0.841). Expression levels of a housekeeping gene, *L13a ribosomal protein* [3], [45], in the pituitary gland did not diverge between forms (ANOVA, *F*_1,25_ = 2.28, *P* = 0.144). Thus, substantial divergence in reproductive hormone signaling exists between spawning females of these two sympatric stickleback species.

### Divergence in male sex steroids

**Figure 5:**
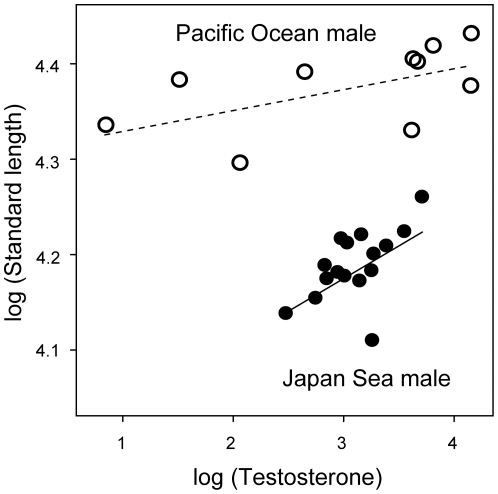
Correlation between log-transformed testosterone levels and log-transformed standard length in males. Solid lines and dotted lines indicate regression lines for Japan Sea form and Pacific Ocean form, respectively. Closed circles, Japan Sea form; open circles, Pacific Ocean form. The slopes of the two regression lines were not significantly different (see the text).

There were no significant differences between nesting males of the two forms in the plasma concentrations of any steroids examined (Fig. 2; 11-ketotestosterone, *F*_1,30_ = 4.37, *P* = 0.052; testosterone, *F*_1,30_ = 0.373, *P* = 0.546; 17β-estradiol, *F*_1,4_ = 0.356, *P* = 0.583), although the Japan Sea males tend to have higher 11-ketotestosterone levels than Pacific Ocean males (Fig. 2A). Interestingly, significant correlations were found between plasma testosterone levels and body length. Within both forms, males with higher testosterone levels were larger in standard length in both forms (Fig. 5; testosterone, *F*_1,23_ = 7.88, *P* = 0.010; form, *F*_1,23_ = 198.65, *P*<0.001; interaction between testosterone and form, *F*_1,23_ = 2.38, *P* = 0.137), whereas no significant correlations between plasma testosterone levels and standard length were found in females (testosterone, *F*_1,33_ = 2.65, *P* = 0.113; form, *F*_1,33_ = 44.40, *P*<10^−6^; interaction between testosterone and form, *F*_1,33_ = 0.09, *P* = 0.772). No other steroids examined were associated with standard length (*P*>0.05).

Because testosterone is a precursor of 11-ketotestosterone (Fig. 1), we investigated the relationships between plasma testosterone and plasma 11-ketotestosterone levels (Fig. 3B). Plasma testosterone level was a significant predictor of plasma 11-ketotestosterone level (ANCOVA, effects of testosterone, *F*_1,14_ = 30.38, *P*<10^−4^). Contrary to what was found in females, 11-ketotestosterone levels were significantly higher in Japan Sea males than in Pacific Ocean males after including testosterone level as a covariate (ANCOVA; effects of form, *F*_1,14_ = 11.24, *P*<0.005: effects of interaction between testosterone and form, *F*_1,14_ = 1.25, *P* = 0.282), suggesting that the efficiency of converting testosterone into 11-ketotestosterone may be higher in the Japan Sea males than in the Pacific Ocean males.

**Figure 6:**
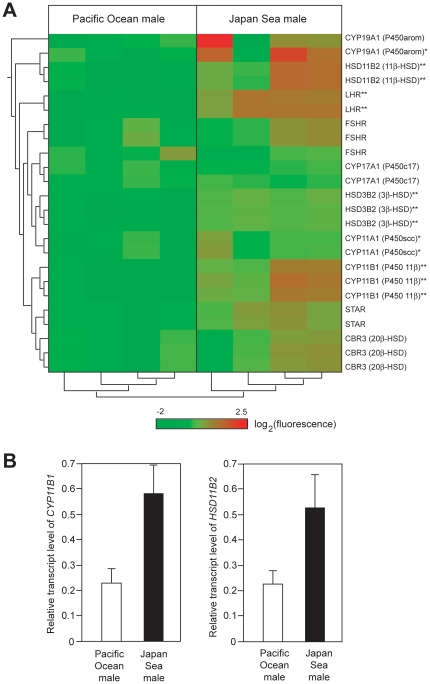
Divergence in the expression levels of steroidogenic enzymes between the gonads of Pacific Ocean and Japan Sea forms. (A) Microarray analysis of testis. Heat map and clustering analysis of the transcripts of genes encoding steroidogenic pathways are shown. Different lines represent different probes. For most genes, multiple independent probes were designed for the same gene. Cluster analysis of probes (shown on the left side of the heat map) indicates that signals of different probes representing the same gene product gave rise to similar signals. Different columns indicate different fish (*n* = 4 males for each form). In the heat map, red colors indicate high fluorescence signals, while green colors indicate low fluorescence signals. Asterisks indicate that the fluorescence signals that were significantly higher in the Japan Sea males than in the Pacific Ocean males by ANOVA; *, *P*<0.05; **, False Discovery Rate-corrected *P*<0.05. (B) qPCR analysis of the transcripts of genes encoding two enzymes involved in the conversion of testosterone into 11-ketotestosterone, *11β-hydroxylase* (*CYP11B1*) and *11β-hydroxysteroid dehydrogenase* (*HSD11B2*) (*n* = 4 fish for each form).

These data further suggest that additional differences in the steroidogenic pathway are likely to exist between forms. We used microarrays to compare the transcriptome of testis, a main sex-steroidogenic organ, between nesting males of the two forms. Although there is great variation between individuals, cluster analysis of samples using transcript levels of mRNA encoding proteins involved in steroidogenic pathways [46] revealed two distinct clusters. One cluster corresponds to the Pacific Ocean form and the other to the Japan Sea form (see the clusters below the heat map in Fig. 6A). These results suggest that there is divergence in the steroidogenic pathways between nesting males of the two forms.

Overall, transcript levels of these genes are higher in the Japan Sea males than in the Pacific Ocean males (Fig. 6A). In particular, the transcripts of genes encoding two enzymes involved in the conversion of testosterone into 11-ketotestosterone, *11β-hydroxylase* (*CYP11B1*) and *11β-hydroxysteroid dehydrogenase* (*HSD11B2*) [46], [47], [48], [49], [50], were more highly expressed in the Japan Sea males than in the Pacific Ocean males (Fig. 6A). This result was also confirmed by quantitative PCR (qPCR): Japan Sea testes had significantly higher transcript levels of *CYP11B1* (ANOVA, *F*_1,7_ = 8.99, *p* = 0.020) and *HSD11B2* (ANOVA, *F*_1,7_ = 7.21, *p* = 0.031) than Pacific Ocean testes. (Fig. 6B). Taken together, these data suggest that the conversion efficiency of testosterone into 11-ketotestosterone may be higher in the Japan Sea males than in the Pacific Ocean males. Interestingly, expression levels of other genes involved in steroidogenesis, such as P450arom (*CYP19A1*), luteinizing hormone receptor (*LHR*), 3β-hydroxysteroid dehydrogenase 2 (*HSD3B2*), and P450scc (*CYP11A1*) were also significantly higher in the Japan Sea males than in the Pacific Ocean males (Fig. 6A). Taken together, these results suggest that there is divergence in steroidogenic pathways between nesting males of the two forms.

## Discussion

Our results demonstrate that two sympatric stickleback species have diverged in sex steroid hormone signaling pathways. First, the patterns of sexual dimorphism in plasma sex steroid levels differ between the sympatric forms. In the Pacific Ocean form, males have higher levels of plasma testosterone than females, whereas levels of plasma testosterone did not show a sex difference in the Japan Sea form. Interestingly, a previous study on a European threespine stickleback population revealed that spawning females had higher concentrations of plasma testosterone concentrations than nesting males [29], [32], [51], which is contrary to what we have found in the Pacific Ocean form. These data demonstrate that there is substantial variation in sexual dimorphism in plasma testosterone levels among threespine stickleback populations. The presence of multiple populations/forms/species that exhibit a variety of patterns of sexual dimorphism in sex steroid levels suggest that the threespine stickleback species complex will be a good model for exploring the genetic mechanisms underlying the evolution of sexual dimorphism in sex steroid levels.

Second, the Japan Sea females had higher plasma sex steroid levels and pituitary *FSHβ* mRNA levels than the Pacific Ocean females. Although pituitary *LHβ* mRNA levels did not differ between the two forms (Fig. 4), transcript levels of the receptor for *LHβ* in the ovary may differ between them. Divergence in the sex steroid signaling pathways may play an important role in the divergence in female reproductive traits between the two forms. Testosterone is known to regulate female mate choice behaviors in humans and birds [10], [52], [53], while17β-estradiol and 11-ketotestosterone are known to regulate female preference behavior in fishes, such as swordtail [54] and the sailfin molly [55]. Sex steroids can also regulate oogenesis and oocyte maturation in fishes [56]. Because the Japan Sea females and Pacific Ocean females diverge in both female mate choice behaviors [26], [36] and fecundity traits [57], further studies including hormonal manipulation experiments [51], [58] should be conducted to investigate the functional roles of sex steroids in female sticklebacks. We have previously found that thyroid hormone signaling pathways diverge between marine and stream ecotypes of threespine stickleback and *cis*-regulatory changes in the *thyroid-stimulating hormone β2* gene are partially contributing to the divergence [3]. By using the genomic tools available for sticklebacks [22], [59], it will be possible to use a similar approach to further investigate the genetic basis of divergence in sex steroid signaling pathways in the Japanese species pair.

Third, male testosterone levels were significantly correlated with male body size within forms, although the plasma testosterone levels were not significantly different between forms. Male body size is important for female mate choice and male-male aggression in the threespine stickleback [60], [61], [62]. Furthermore, body size divergence is involved in behavioral isolation between sympatric species, including the Japanese sympatric pairs [60], [61], [62]. Because body size is involved not only in behavioral isolation, but also in ecological divergence, body size is considered as a potential “magic trait” in sticklebacks [60], [61], [62], [63]. Although testosterone is known to regulate skeletal development in many animals [64], [65], [66], we currently do not know whether variation in testosterone level is the cause or the consequence of variation in body size. It is also possible that differential expression patterns of androgen receptors rather than plasma levels of testosterone contribute to phenotypic divergence [67]. Therefore, further studies on the link between testosterone signaling and male body size are required for a better understanding of the contribution of hormonal regulation to traits involved in ecological adaptation and reproductive isolation.

Intra-specific variation in sex steroids is known to regulate reproductive polymorphisms across diverse taxa [54], [68], [69], [70], [71], [72]. By contrast, little is known about the role of divergence in sex steroid signaling in speciation. Our results demonstrate that divergence in sex steroid signaling pathways can occur between incipient species breeding in sympatry. Sex steroids not only regulate the expression of reproductive traits, but also of other physiological traits, such as salinity tolerance [73], migratory behaviors [74], and feeding behaviors [75], [76], [77]. Therefore, we suggest that it is crucial to investigate the genetic and ecological mechanisms of divergence in hormonal signaling between incipient sympatric species for a better understanding of speciation mechanisms in animals. Furthermore, because endocrine disruptors can affect sex steroid signaling [78], it is important to investigate how water pollution with endocrine disruptors will influence the patterns of reproductive isolation between incipient species in nature.

## Materials and Methods

### Ethics Statement

Animal use protocols were approved by the Institutional Animal Care and Use Committee of the Fred Hutchinson Cancer Research Center (1575) and the National Institute of Genetics (23-15).

### Plasma collection

Because sex steroid levels can vary among different reproductive stages [31], our present study focused exclusively on nesting males and spawning females; samples were collected immediately after mating experiments were conducted in the laboratory. Sympatric Pacific Ocean and Japan Sea sticklebacks were collected from the Bekanbeushi River System on Hokkaido Island, Japan [26], [36], [37], [79], in May and June of 2006–2008. Mating experiments were conducted in June–July, which is the peak of breeding season for these two sticklebacks [36], [79]. We conducted two sets of experiments. In the first set of experiments, one male was mated with one female of the same species (no-choice experiment) as described previously [26], [36]. Briefly, a single breeding male was put into a nesting tank. Once the male made a nest, a gravid female of the same form was put into the same tank. Immediately after the female inspected the nest, both male and female were taken out of the tank prior to spawning. After immersion of the fish in a lethal dose of MS222, blood was collected from the caudal tail and centrifuged at 3,000 g for 10 min to isolate the supernatant (plasma) [3].

In the second set of experiments, one female was allowed to choose one of two males, either a Japan Sea male or a Pacific Ocean male (female mate choice experiment) as described previously [26], [36]. Briefly, one large tank was divided into two compartments, each of which contained one Japan Sea male and one Pacific Ocean male. Once both males made nests, a single gravid female was put into a small transparent box located between the two compartments so that the female could see both males. Fifteen minutes later, the female was released into the tank and behaviors were monitored until the female inspected one of the nests. Blood was immediately collected from the female. Blood was also collected from the nesting males after the behavioral trial. Each male was used for testing with one Japan Sea female and one Pacific Ocean female. All plasma was stored at −70°C until assay. Standard length was measured with a vernier caliper immediately after the behavioral experiments.

### Measurement of sex steroids

Plasma concentrations of testosterone and 17β-estradiol were measured with radioimmunoassay at the Center of Reproductive Biology, Washington State University, Pullman, Washington, USA. Plasma concentrations of 11-ketotestosterone were measured with enzyme-linked immunosorbent assay at the Northwest Fisheries Science Center, Seattle, WA, USA. Because only small amounts (usually less than 20 µl) of plasma could be collected from a single fish, concentrations of all steroids could not be measured from every fish, so sample sizes vary between steroids. In addition, plasma concentrations of 11-ketotestosterone were lower than the detection threshold in 5/14 Pacific Ocean females, which is consistent with our conclusion that Pacific Ocean females have the lowest plasma 11-ketotestosterone levels (Fig. 2A). Plasma concentrations of 17β-estradiol were lower than the detection threshold in 1/23 Japan Sea females, 2/20 Pacific Ocean females, 12/14 Japan Sea males, and 7/11 Pacific Ocean males, which is consistent with our conclusion that females have higher plasma 17β-estradiol levels than males (Fig. 2B). Plasma concentrations of testosterone were lower than the detection threshold in 1/20 Pacific Ocean females, which is consistent with our conclusion that Pacific Ocean females have the lowest plasma testosterone levels (Fig. 2C). These samples were excluded from the analyses, so our statistical tests are rather conservative.

Although 17β estradiol levels differed significantly between choice and no-choice experiments in the Pacific Ocean females (means ± s.e. of plasma 17β-estradiol were 2.75±0.75 ng/ml and 0.47±0.13 ng/ml for the choice and no-choice experiments, respectively; ANOVA, *F*_1,15_ = 12.9, *p* = 0.0026), other sex steroid levels did not differ between choice and no-choice experiments in males or females of either form (ANOVA, *P*>0.05). Even in the case of Pacific Ocean female 17β estradiol, inclusion of the type of behavioral trial as a factor did not change our conclusions, so we pooled data obtained from both choice and no-choice experiments for investigating the overall patterns of variation in sex steroid levels in the Japanese species pair.

### Quantitative PCR

After behavioral experiments, pituitary glands were collected from spawning females (*n* = 19 for Japan Sea females and *n* = 8 for Pacific Ocean females) and gonads were collected from nesting males (*n* = 5 for Japan Sea males and *n* = 4 for Pacific Ocean males). Each tissue from each fish was stored separately in a Non-stick RNase-Free Microcentrifuge Tube (Ambion, Austin, TX, USA). Pituitary total RNA was isolated with RNeasy Micro Kit (Qiagen, Valencia, CA, USA), while testis total RNA was once isolated with Trizol Reagent (Invitrogen, Carlsbad, CA, USA) and then purified again with the RNeasy Micro Kit. Pituitary RNA (50 ng) was reverse transcribed to cDNA with High-Capacity cDNA Reverse Transcription Kits (Applied Biosystems, Foster City, CA, USA). RNA from each tissue from each fish was treated separately and never pooled to maximize the number of biological replicates.

Primers were designed with Primer Express software (Applied Biosystems, Foster City, CA, USA): for *LHβ* (GenBank accession number AJ534969; [44]), forward primer (5′-GACGGCTCTTCAGGTCAGAAA-3′) and reverse primer (5′-GAAAGTCGAGGCTCCCAGAA-3′) were used; for *FSHβ* (GenBank accession number AJ534871; [44]), forward primer (5′-GGGTGCCAAGAGAACAGCTTCA-3′) and reverse primer (5′-TGGTGTGGATGGACGACGTGTTT-3′) were used; for *11β-hydroxylase* (Ensembl Transcript ID ENSGACT00000015449), forward primer (5′-TGCCGAGAACGAGATGCA-3′) and reverse primer (5′-GGACAACACGCTGAGATGGA-3′) were used; for *11β-hydroxysteroid dehydrogenase* (Ensembl Transcript ID ENSGACT00000023181), forward primer (5′-CAGGATGTGACTCTGGTTTTGG-3′) and reverse primer (5′-GAACACCTCGAAGCCGAGATT-3′) were used. These primer pairs gave rise to a single peak in the dissociation curve, suggesting that the primer dimer or PCR by-products are minimal. We also conducted qPCR with a control housekeeping gene, *L13a ribosomal RNA gene*, as described previously [3], [45]. The KAPA SYBR FAST qPCR kit (KAPA Biosystems, Boston, MA, USA) was used for qPCR reaction. For pituitary gland samples, qPCR was run on ABI PRISM 7900 HT (Applied Biosystems, Foster City, CA, USA). For testis samples, qPCR was run on ABI Step One Plus (Applied Biosystems, Foster City, CA, USA). Relative expression levels were calculated from the standard curves, which were drawn from serially diluted cDNA pools of all analyzed fish.

### Microarray experiments

Microarray experiments were conducted as described previously with several modifications [3]. Testes were collected from four nesting Japan Sea males and four nesting Pacific Ocean males. Total RNA was isolated with Trizol Reagent (Invitrogen, Carlsbad, CA, USA), followed by purification with RNeasy Micro Kit (Qiagen, Valencia, CA, USA). RNA from each fish (*n* = 8) was labeled with Cy3 and hybridized separately to a custom-made microarray (*n* = 8 arrays) created by Agilent Technologies (Santa Clara, CA, USA). RNA from different fishes was not pooled, so there are four biological replicates of each form. In addition to the 43,392 unique oligonucleotide probes representing 19,274 genes [80], we added new 4617 unique probes representing 3950 genes that were newly uploaded in the newer version of the Ensembl database (Release 60; http://nov2010.archive.ensembl.org/index.html). The probe sequences and data are deposited at the Center for Information Biology Gene Expression (CIBEX) database (http://cibex.nig.ac.jp/index.jsp) (CIBEX accession number CBX247).

The experiments and data normalization were conducted by DNA Chip Research Institute (Yokohama, Kanagawa, Japan), as described previously [3]. Briefly, the arrays were hybridized with Cy3-labeled RNA and scanned with the Agilent DNA microarray scanner (Agilent Technologies, Santa Clara, CA, USA). In order to exclude differences in signal intensity among arrays, signals were first normalized to the 75th percentile of each array. In order to exclude the difference between probes, the signals were log-transformed with base 2 and then normalized by dividing each value by the median of each probe.

Clustering was conducted with CLC Genomics Workbench (CLC bio, Katrinebjerg, Denmark) based on an Euclidean distance matrix. For clustering analysis of genes involved in steroidogenesis, we used mRNA signals encoded by the following genes [46]; *LH receptor* (*LHR*; ENSGACG00000005554), *FSH receptor* (*FSHR*; ENSGACG00000002728), *steroidogenic acute regulatory protein* (*STAR*; ENSGACG00000011782), *11β-hydroxylase* (*CYP11B1*; ENSGACG00000011657), *3β-hydroxysteroid dehydrogenase 2* (*HSD3B2*; ENSGACG00000001425), *P450scc* (*CYP11A1*; ENSGACG00000004713), *P450c17* (*CYP17A1*; ENSGACG00000002550), *aromatase* (*CYP19A1*; ENSGACG00000016742), *20β* -*hydroxysteroid dehydrogenase* (*CBR3*; ENSGACG00000011333), and *11β-hydroxysteroid dehydrogenase 2* (*HSD11B2*; ENSGACG00000017514). Heat maps were drawn with the CLC Genomics Workbench. Statistical tests of microarray data were also conducted with CLC Genomics Workbench.
